# Influence of exogenous corticosterone on testicular function and mating behavior of Nigerian indigenous cocks

**DOI:** 10.1590/1984-3143-AR2021-0026

**Published:** 2022-03-11

**Authors:** Oluwaseun Serah Iyasere, Temitope Ayisat Ajadi, Richard Abayomi Sobayo, Mariam Opeyemi Logunleko, Adenrele Olalekan Adebayo, Samuel Olutunde Durosaro, Lawrence Tokunbo Egbeyale, Oluwabukunmi Oluwayemisi Famosaya, Oluwatosin Olawanle Ajiboye, Sakiru Oladele Akinbode, James Olamitibo Daramola

**Affiliations:** 1 Department of Animal Physiology, Federal University of Agriculture, Abeokuta, Ogun State, Nigeria; 2 Department of Veterinary Public Health and Reproduction, College of Veterinary Medicine, Federal University of Agriculture, Abeokuta, Ogun State, Nigeria; 3 Department of Animal Nutrition, Federal University of Agriculture, Abeokuta, Ogun State, Nigeria; 4 Department of Veterinary Anatomy, Federal University of Agriculture, Abeokuta, Ogun State, Nigeria; 5 Department of Animal Breeding and Genetics, Federal University of Agriculture, Abeokuta, Ogun State, Nigeria; 6 Department of Animal Production and Health, Federal University of Agriculture, Abeokuta, Ogun State, Nigeria; 7 Department of Economics, College of Management Sciences, Federal University of Agriculture, Abeokuta, Ogun State, Nigeria

**Keywords:** chickens, hormones, mating behavior, semen quality, testes

## Abstract

In bridging the knowledge gap on stress physiology of Nigerian indigenous chickens, this study investigated the effect of exogenous corticosterone (eCORT) as stress inducing agent on the testicular function and mating behavior of Nigerian indigenous cocks. Twenty-four (24) cocks and one hundred and forty four (144) hens (mating ratio of 1 cock: 6 hens) were grouped into four and assigned to each of the four eCORT treatments (0, 2, 4 and 6 mgeCORT/KgBW) daily for 14 days. Semen samples were collected on days 0, 7 and 14 and analyzed for semen volume (SV), progressive sperm motility (PSM), membrane integrity (MI) and sperm abnormality (SA). Mating behaviors were monitored on days 3, 5 and 8. Blood samples, for hormonal (Luteinizing Hormone (LH), Follicle Stimulating Hormone (FSH) Testosterone (TEST) and stress analysis (heterophil/lymphocyte ratio, H/L) were collected from brachial vein on days 7 and 14. On day 15, cocks were euthanized and testes harvested for histomorphometry. Data were analyzed using multivariate analysis, one–way ANOVA and Kruskal-Wallis tests all in SPSS 23. Administration of 4 mgeCORT/KgBW declined (P<0.05) PSM while 4 mgeCORT/KgBW and 6 mgeCORT/KgBW cocks had reduced (P<0.05) SV and MI with increased SA. Compared to baseline values, progressive sperm motility of cocks administered 6 mgeCORT for 7 and 14 days decreased (P*<*0.05) by 57.5% and 52.4%, respectively. Exogenous CORT had no significant (P>0.05) influence on the mating behaviors, H/L ratio, FSH and TEST. However, 2 mgeCORT/KgBW enhanced LH levels. Administration of eCORT did not affect the testicular epithelial height and seminiferous tubular diameter. In conclusion, optimal stress induced by eCORT impaired semen quality but with less impact on reproductive hormones, H/L and mating behaviors of intensively raised Nigerian indigenous cocks.

## Introduction

Nigeria indigenous chickens according to Resources Inventory Management (Federal Department of Livestock and Pest Control Services, 1992) represent 80% of the 120 million poultry types reared in the rural areas of Nigeria. They are important in the overall food production systems (Adeoye et al., 2017). Their products serve as a good source of income generation for rural people and are also an important source of high quality protein for developing countries (Matawork 2018). The effect and responses to stress differ from one species to another with Nigerian indigenous chicken being reported to have high degree of adaptability to prevailing condition (Ige, 2013). While there are reports on stress physiology and reproduction of the exotic chickens, there is paucity of information on the indigenous breeds.

Generally, in response to stress, glucocorticoids (e.g. corticosterone) play a vital role by producing an array of effects on body functions. According to Pilo et al. (1985), administration of exogenous corticosterone caused gonadal regression in chickens. In addition, exposure of chickens to corticosterone caused deterioration in most reproductive performance of male domestic fowls (Eid et al., 2006) and laying hens (Shini et al., 2009). Alteration of steroidogenesis and gametogenesis sequel to change in glucocorticoid levels has also been well documented (Whirledge and Cidlowski, 2010). Besides, in some studied birds, this change equally affects semen qualities as well as sexual behaviors. A significantly positive correlation was reported between sperm concentration and time of sexual libido in quails (Hanafy and Khalil, 2015).

Although, the effect of stress and corticosterone (CORT) on the reproductive performance in domestic chickens have been well established (Carsia and Harvey, 2000), reports on the effect of CORT on testicular functions of Nigerian indigenous cocks are scarce. While the indigenous chickens are believed to be hardy with the ability to thrive under harsh environmental conditions, its low productivity may not be unconnected with the dearth knowledge on the effect of stress on its reproductive function. Therefore, this work examined the influence of exogenous corticosterone (eCORT) on testicular function and mating behavior of the Nigerian indigenous cocks. We tested the hypothesis that Nigerian indigenous cocks cannot maintain their hardiness when corticosterone is elevated for 14 consecutive days and so their semen quality, testicular histomorphometry, mating behavior and reproductive hormone will be affected.

## Methods

### Animals and housing

The study was carried out at the Poultry Unit of Teaching and Research Farm and the laboratory of Animal Physiology Department, all in the Federal University of Agriculture, Abeokuta (FUNAAB; 7° 10' N and 3° 2' E and 76 m above sea level). The experiment began in August and ended in December 2018. The poultry house was open sided, so birds were exposed to natural light (12L: 12D) and daily fluctuation of temperature and humidity.

12 months old Nigerian indigenous chickens (*n* = 24; cocks of average weight of 1100 ± 95.5 g and *n* = 144; hens of average weight 800 ± 44.8 g) raised in the Poultry Unit of Teaching and Research Farm (FUNAAB) were used for this study. The birds were habituated for two weeks and were treated against endo-parasite and ecto-parasites. They were also trained for semen collection using abdominal massage technique described by Okoro et al. (2016). To simulate the natural social environment, one cock was housed with 6 hens in each pen. Birds were housed in deep litter floors pens covered with 5 cm wood shaving (2m×5m, giving a stocking density of 590 g/m^2^). Other descriptions of the poultry house are the same as that reported by Iyasere et al. (2020). Birds were fed layers mash concentrate feed (16.5% CP and 2725-2980 kcal/kg metabolizable energy) at the rate of 120 g/bird/day (recommended quantity for laying bird), to prevent excess fat accumulation in the hens which could affect laying. Water was provided for *ad libitum* consumption. Three wooden nest boxes (30cmx30cmx50cm) were also provided in each pen for the hens to perform their nesting and egg laying activities.

### Corticosterone delivery and data collection

After the habituation period, cocks were randomly assigned to four exogenous corticosterone (eCORT) treatments namely 0, 2, 4 and 6 mgeCORT/KgBW daily for 14 days. Each treatment had six replicates and each replicate had 1 cock (6 cocks per treatment). The eCORT (sigma-Aldrich, USA) was first dissolved in ethanol and then made up with distilled water to 1mg/ml concentration. Cocks were gently restrained and their respective eCORT dosage were administered via gavage twice a day (half doses of 0, 1, 2 and 3 mgeCORT/KgBW in the morning (08:00-09:00) and the remaining half dose in the evening (16:00-17:00) to mimic the natural surges of CORT when exposed to daily stressors. Control cocks were administered only with 2ml distilled water. To collect semen samples, cocks were separated into an individual cages 24-h ahead and semen were collected from each cock on day 0, 7 and 14 of eCORT administration. Blood sampling for reproductive hormone analysis was collected on the 7 and 14^th^ day. Mating behavior of the cocks was recorded on the 3^rd^, 5^th^ and 8^th^ day of eCORT administration using CCTV camera mounted in different angles in the pen. After the 14^th^ day of treatment, the cocks were euthanized and their testes dissected out for histomorphometric analysis.

### Evaluation of semen quality

Semen volume (SV) was measured using a graduated eppendoff tube and read directly to the nearest 0.01 ml. Progressive sperm motility (PSM) was determined as described by Bearden and Fuquay (1997). Briefly, 5 μl semen sample was dropped on a pre-warmed microscope slide (37 ^o^C) overlaid with a 22 x 22 mm cover slip and viewed at 400x magnification using Celestron-PentaView LCD digital microscope (44348 model by Celestron, Italy). Ten microscopic fields were examined for each sample to determine progressive sperm motility and the mean of the ten successive evaluations was expressed as the overall percentage of progressive sperm motility.

Sperm concentration (SC) was determined according to Dascanio (2014) technique and Beckmam spectrophotometer sperm calibration. 15 μl of each semen sample was added to 3 ml of 2.9 % sodium citrate solution in a cuvette. The mixture was gently mixed and placed in the cuvette holder of the UV spectrophotometer (SW7504 model by Surgifriend Medicals, England). The absorbance (A) was read at 650nm wavelength for each sample. Sperm concentration (x10^6^/ml) was calculated as;


$$11,170\;X\;\left(A\right)\;–\;90$$ (1)

Where; 

A is the absorbance read by the spectrophotometer

Membrane integrity (MI) was determined using the Hypo-Osmotic Swelling Test (HOST) as described by Zubair et al. (2013). To do this, Ten (10 μl) of semen sample was incubated in 100 μl hypoosmotic solution (9 g fructose plus 4.9 g sodium citrate mixed with 1000 ml of distilled water) at 37 ^o^C for 30 min, 0.1 ml of the mixture was spread over pre-warmed slide, covered with a cover slip and observed at 400x magnification with Celestron Penta View LCD digital microscope (44348 model by Celestron, Italy). Ten microscopic fields were assessed for spermatozoa swelling characterized by coiled tail which indicate an intact plasma membrane.

Sperm abnormalities (SA) were determined as described by Bearden and Fuquay (1997) using eosin-nigrosin stain. Briefly, on a pre-warmed slide, 3 μl of semen was stained with 2 μl eosin-negrosin. Defects in the head, midpiece and tail of sperm cells were observed for five microscopic fields at 400x magnification using Celestron PentaView LCD digital microscope. The total abnormality (%) was obtained as the mean of the five microscopic observations per slide.

### Observation of mating behavior

Behavior of the cocks was monitored using a Closed-Circuit Television (CCTV) Cameras (CP PLUS) mounted in each pen and connected to Digital Video Recorder (DVR) for recording and data storage into a hard drive. Mating behavior of each cock was observed in the morning (09:00-11:00) and evening (17:00-18:30) on days 3, 5 and 8 of the eCORT administration. Ethogram showing attempted mounts, successful mating and Waltz behaviors is presented in Table 1. Behavioral data was extracted from the video playback using the Behavioral Observation Research Interactive Software (BORIS), the behavior in Table 1 were coded as an “event based” into the BORIS software. Extracted data such as frequency, mean duration and total duration of waltzing, attempted mount and successful mating were exported to Microsoft excel sheets where the data were arranged appropriately for statistical analysis.

**Table 1:** Description of mating behavior.

**Behavior**	**Description**
Waltz	Approach of a male to a female while the male drops a wing to the ground and performing a semi-circular movement around the hen
Attempted mounts	Cock makes an attempt to mount the female for mating process, usually including grabbing the hen at the back of the neck behind the head or comb with its beak
Successful mating	Cocks successfully completed copulation i.e. cloaca contact between cock and hen

Source: Moyle et al. (2010).

### Collection and analysis of blood

To assess reproductive hormones, 2 ml of blood samples were collected from the brachial vein of the cocks into EDTA containing bottles using sterile syringes on days 7 and 14 of eCORT administration between 10:00 and 12:00 hrs. Testosterone (TEST), follicle stimulating hormone (FSH) and luteinizing hormone (LH) were measured in one time analysis using ELISA kits (Bio-Inteco Diagnostics, UK) specific for each hormone and the absorbance were read using ELISA reader (LX800). For heterophil and lymphocyte ratio, blood samples were smeared and air dried after which they were stained with May-Grunwald-Giesma stain as described by Robertson and Maxwell (1990). The number of heterophil and lymphocyte were counted under microscope to a total of 100 cells. The result was expressed as a percentage and H/L ratio was obtained by dividing heterophils number by lymphocytes number (Gross and Siegel 1983).

### Histomorphometry procedure

Histological examination of the testes was carried out as described by Zahid et al. (2002). The testicles were dissected out and tissue samples of the testes were fixed in fresh Bouin’s solution for 72 hrs, and then subjected to routine histological procedures: dehydration in ascending alcohol concentrations, clearing with xylene, embedding in paraffin, cutting on a Reichert microtome, mounting of 5 µm thick sections on a glass slide. Each section was stained with haematoxylin-eosin using standard staining procedures according to Luna (1968). Measurement of testicular histomorphometry was performed under light microscope using am-scope camera attached to a laptop. The measure of the tubular diameters was carried out through vertical, horizontal and diagonal measurement of four seminiferous tubules (round or nearly round) randomly chosen from each pair of testis. Vertical, horizontal and diagonal measurements of the epithelial height (EH) were made for each of the four seminiferous tubules considering the height of the basal laminar of the seminiferous tubules to the lumen. The average tubular diameter and epithelial height of the four seminiferous tubules were calculated and were considered as the seminiferous tubule diameter (STD) and epithelial height for each pair of the testis (Weibel et al.*,* 1966).

### Statistical analysis

All analyses were carried out using SPSS version 23. Data obtained on semen quality were subjected to General Linear Model (GLM) considering trial day (0, 7 and 14) and dose of eCORT (0, 2, 4 and 6 mgeCORT/KgBW) as fixed factors. Testicular histomorphometric data were subjected to One-way ANOVA. Data on mating behavior which satisfied Shapiro-Wilk normality test (frequency of attempted mounting, frequency of successful mating, mean duration of attempted mounting and mean duration of successful mating) were analyzed using One-way ANOVA while those which failed the normality test (frequency of waltzing, total duration of waltzing, mean duration of waltzing, total duration of attempted mounting and total duration of successful mating) were analyzed using non-parametric test (Kruskal-Wallis test). Hormones/blood data for 7 and 14 days of eCORT administration were analyzed using a One-way ANOVA. Turkey’s post hoc test was performed to investigate significant differences (p*<*0.05).

### Ethical approval

This research was conducted in accordance with the Institutional Animal Ethics Committee guidelines of the Federal University of Agriculture, Abeokuta, Nigeria with approval number FUNAAB/AEWC/2020/0021. The guidelines for animal care were carried out in accordance with the UK Animals (Scientific Procedures) Act, 1986 and association guidelines of the Nigerian Institute of Animal science (NIAS)

## Results

### Effect of eCORT on semen quality parameters of Nigerian indigenous cocks

Semen quality of Nigerian indigenous cocks are influenced by the different doses of eCORT administered (Table 2). SV and PSM was lowest (P*<*0.05) in 4 mgeCORT/KgBW cocks followed by 6 mgeCORT/KgBW, MI decreased significantly (P*<*0.05) in cocks administered 4 mgeCORT/KgBW and 6 mgeCORT/KgBW. SA increased (P=0.013) with increasing doses of eCORT administered but SC was not significantly (P>0.05) affected by eCORT. The effect of days of eCORT administration is shown in Table 3. SV, PSM and MI declined significantly (P*<*0.05) with increasing days of administration of eCORT, SA increased (P=0.000) as the day of eCORT administration increased while SC was not affected (P>0.05). Table 4 shows the interactive effect of dose and day of administration of eCORT on semen quality of Nigerian indigenous cocks. Cocks administered with 4 mgeCORT/KgBW and 6 mgeCORT/KgBW had reduced SV at day 14 (P=0.006). Also, PSM of cocks administered 6 mgeCORT/KgBW for 7 and 14 days decreased by 57.5% and 52.4% respectively. There was no significant (P>0.05) interaction between dose of eCORT and day of administration on SC, MI and SA.

**Table 2:** Main effect of exogenous corticosterone (eCORT) on semen quality of Nigerian indigenous cocks.

**eCORT/KgBW**	**SV (ml)**	**PSM (%)**	**SC (x10^6^/ml)**	**MI (%)**	**SA (%)**
0 mg	0.15±0.02^a^	67.44±4.39^a^	1264.11±125.87	45.61±3.06^a^	7.84±1.32^b^
2 mg	0.15±0.03^a^	62.53±6.53^a^	1197.53±148.42	43.63±3.79^a^	12.16±1.45^ab^
4 mg	0.07±0.01^b^	38.11±4.85^b^	1113.88±197.15	33.22±3.13^b^	14.31±2.43^ab^
6 mg	0.13±0.02^ab^	56.61±6.13^a^	1124.82±132.14	33.94±4.54^b^	16.63±3.19^a^
P-value	0.014	0.000	0.888	0.018	0.013

^a,b^Means with different superscript differs significantly (*P<*0.05), values are Means ±SE. SV = semen volume, PSM = progressive sperm motility, SC = semen concentration, MI = membrane integrity, SA = sperm abnormality

**Table 3:** Main effect of day of eCORT administration on semen quality of Nigerian indigenous cocks.

**Day of eCORT administration**	**SV (ml)**	**PSM (%)**	**SC (x10^6^/ml)**	**MI (%)**	**SA (%)**
0 day	0.16±0.02^a^	74.64±4.66^a^	1419.92±178.21	44.84±3.96^a^	6.34±1.10^b^
7 day	0.11±0.01^b^	47.33±4.19^b^	1002.38±104.83	43.00±2.73^a^	14.30±1.77^a^
14 day	0.11±0.02^b^	46.85±4.71^b^	1102.96±77.11	29.54±2.27^b^	17.56±2.37^a^
P-value	0.040	0.000	0.081	0.001	0.000

^a,b^Means with different superscript differs significantly (*P<*0.05), values are Means ±SE. SV = semen volume, PSM = progressive sperm motility, SC = semen concentration, MI = membrane integrity, SA = sperm abnormality

**Table 4:** Interactive effect of eCORT and days of administration (Day) on semen quality of Nigerian indigenous cocks.

**eCORT/KgBW**	**Day**	**SV (ml)**	**PSM (%)**	**SC (×10^6^)**	**MI (%)**	**SA (%)**
0 mg	0	0.13±0.03^abc^	75.50±4.54^abc^	1518.47±249.69	44.33±6.34	4.57±1.43
	7	0.12±0.02^abc^	58.50±10.06^abcde^	1053.26±221.51	54.67±4.36	9.23±2.54
	14	0.20±0.04^ab^	68.33±6.72^abcd^	1220.61±167.29	37.83±2.77	9.73±2.46
2 mg	0	0.18±0.07^abc^	84.20±6.94^ab^	1245.93±346.35	54.20±7.25	9.92±3.42
	7	0.13±0.05^abc^	55.00±11.92^abcde^	1138.70±284.67	44.00±4.32	13.88±1.87
	14	0.12±0.04^abc^	48.40±8.74^bcde^	1207.95±169.74	33.00±4.83	12.68±2.18
4 mg	0	0.08±0.01^bc^	48.33±10.54^bcde^	1337.89±544.97	35.00±7.63	3.90±1.67
	7	0.09±0.02^abc^	37.83±4.35^de^	894.82±224.54	39.33±2.97	15.20±2.44
	14	0.04±0.01^c^	28.17±8.29^e^	1108.91±171.48	25.33±3.54	23.83±3.42
6 mg	0	0.23±0.03^a^	89.33±2.51^a^	1577.36±288.34	45.83±9.83	6.97±1.92
	7	0.09±0.02^abc^	38.00±3.41^de^	922.75±157.06	34.00±6.27	18.90±5.34
	14	0.06±0.01^bc^	42.50±6.51^cde^	874.34±92.90	22.00±4.92	24.01±6.41
P-value		0.006	0.054	0.922	0.467	0.156

^a,b,c,d,e^Means with different superscript differs across the rows, (*P*<0.05), values are Mean ±SE. SV = semen volume, PSM = progressive sperm motility, SC = semen concentration, MI = membrane integrity, SA = sperm abnormality.

### Effect of eCORT on testicular histomorphometry of Nigerian indigenous cocks

The histomorphometry of the cock testes is shown in Table 5. At the end of the 14^th^ day of eCORT administration, the seminiferous tubules diameter and epithelial height were not significantly affected by eCORT.

**Table 5:** Effect of 14 days eCORT on testicular histomorphometry of Nigerian indigenous cocks.

**eCORT/KgBW**	**Epithelilal Height (μm)**	**Seminiferous Tubules Diameter (μm)**
0 mg	82.90±3.77	227.34±5.67
2 mg	79.15±5.27	234.09±7.57
4 mg	83.57±2.22	244.50±5.89
6 mg	83.21±2.10	242.41±5.02
P-value	0.803	0.228

Values are Means ±SE.

### Effect of eCORT on mating behavior of Nigerian indigenous cocks

The effect of eCORT on mating behavior of Nigerian indigenous chickens is presented in Table 6 and Table 7. Exogenous CORT had no effect (P>0.05) on frequency of attempted mounts, frequency of successful mating, mean duration of attempted mounts and mean duration of successful mating of Nigerian indigenous cocks (Table 6). The frequency of waltzing, total duration of waltzing and mean duration of waltzing was not significantly affected by eCORT. Total duration of attempted mating and total duration of successful mating were also not influenced by eCORT (Table 7).

**Table 6:** The effect of eCORT on frequency and mean duration of attempted and successful mating of Nigerian indigenous chickens.

**eCORT/KgBW**	**FAM**	**FSM**	**MDAM (min)**	**MDSM (min)**
0 mg	1.50±1.55	0.97±1.05	0.87±0.44	2.13±1.70
2 mg	1.25±0.73	0.78±0.54	0.96±0.30	1.54±1.02
4 mg	1.19±0.95	0.81±0.69	0.86±0.45	1.38±1.13
6 mg	1.70±1.25	0.94±0.77	0.90±0.49	2.21±1.77
P-value	0.868	0.963	0.978	0.685

FAM = frequency of attempted mounts, FSM = frequency of successful mating, MDAM = mean duration of attempted mounts and MDSM = mean duration of successful mating, values are Mean ± SE.

**Table 7:** The effect of eCORT on waltzing behavior of Nigerian indigenous chickens.

**eCORT/KgBW**	**FWLT**	**TDWLT (min)**	**MDWLT (min)**	**TDAM (min)**	**TDSM (min)**
0 mg	15.25	16.75	16.92	12.00	13.42	
2 mg	12.50	11.25	10.67	12.50	12.50
4 mg	12.50	12.58	12.67	11.67	10.42
6 mg	9.75	9.42	9.75	13.63	13.67
P-value	0.059	0.317	0.297	0.955	0.853

FWLT = frequency of waltzing, TDWLT = total duration of waltzing, MDWLT = mean duration of waltzing, TDAM = total duration of attempted mating, TDSM = total duration of successful mating, Values are Mean Ranks.

### Effect of eCORT on H/L ratio and reproductive hormone of Nigerian indigenous cocks

Table 8 shows the effect of eCORT administration for 14 days on H/L ratio and plasma reproductive hormone of Nigerian indigenous cocks. After 7 days of eCORT, there was no significant (P>0.05) difference in H/L, FSH, LH and TEST concentration. However, after 14 days of administration, eCORT had a significant effect (P*<*0.05) on LH being greater in cocks administered 2 mgeCORT/KgBW. FSH and TEST remained insignificantly (P>0.05) influenced by eCORT after 14 days.

**Table 8:** Effect of eCORT administration on H/L, FSH, LH and TEST of Nigerian indigenous cocks.

**eCORT/KgBW**	**H/L ratio**	**FSH (mIU/ml)**	**LH (mIU/ml)**	**TEST (ng/ml)**
Day 7				
0 mg	0.542±0.556	3.864±0.190	11.10±2.98	0.316±0.071
2 mg	0.528±0.061	3.633±0.199	8.82±1.15	0.253±0.076
4 mg	0.515±0.063	6.240±1.07	16.60±4.10	0.293±0.074
6 mg	0.557±0.091	5.770±2.39	10.37±3.04	0.213±0.073
P-value	0.977	0.450	0.301	0.799
Day 14				
0 mg	0.57±0.062	3.70±0.057	7.932±0.509^b^	0.257±0.051
2 mg	0.446±0.044	3.987±0.466	13.050±2.047^a^	0.298±0.083
4 mg	0.400±0.045	3.278±0.152	7.997±0.906^b^	0.220±0.064
6 mg	0.462±0.041	4.185±0.171	8.0117±0.343^b^	0.398±0.103
P-value	0.073	0.112	0.011	0.420

^a,b^Means with different superscript differ significantly (P<0.05) at day 14 for LH, values are Means ±SE. H/L= heterophil/lymphocyte ratio, FSH= Follicle stimulating hormone, LH= Luteinizing hormone, TEST=Testosterone

## Discussion

This study investigated the impact of exogenous CORT (eCORT) for 14 days to mimic stress conditions on the testicular function and mating behavior of Nigerian indigenous cocks. The decline in semen volume and progressive sperm motility coupled with an increased sperm abnormality observed in the current study might be an indication of the damaging effects of eCORT on semen quality of Nigerian indigenous cocks. Cocks administered with eCORT in this study had decreased semen volume with continuous administration of high dose of CORT especially 4 mgCORT/KgBW and 6 mgCORT/KgBW. Semen volume may not directly correlate with viability of the sperm. However, it is evaluated because seminal fluids serve as a medium of transportation for the sperm cells (Adeoye et al.*,* 2017). Deviche et al. (2011) reported that the spermatogenic cycle of cocks is 13-14 days and cocks administered with eCORT had reduced semen volume and progressive sperm motility after 7 and 14 days of continuous administration. This implies that corticosterone had effect on stored spermatozoa in the ductus deferens since this is the major storage organ for spermatozoa in birds (Moyle et al.*,* 2011). These findings are in agreement with the results obtained by Hanafy and Khalil (2015) where the administration of dexamethasone (synthetic glucocorticoid) at 0.25 and 0.50 mg/bird for 14 successive days significantly decreased sperm motility and live-dead ratio. Similar result has also been reported by Eid et al. (2006) where sperm motility was impaired and percentage of dead sperm cells increased in cocks following the injection of 4 mg dexamethasone/bird/day for 7 days continuously. Spermatozoa, once deposited inside the female reproductive tract strive to reach the oocyte first and acquire hyper-activated progressive motility regardless of linear speed (WHO, 2010). A high motility of spermatozoa thereby becomes necessary at the fertilization site to reach and penetrate the oocyte (Langendijk et al.*,* 2002).

Sperm membrane integrity is an indicator of sperm vitality and is necessary in order to maintain normal sperm function (López, 2012). In this study, decreased membrane integrity was recorded in cocks administered 4 and 6 mgeCORT/KgBW. The sperm membrane integrity obtained in this study is similar to 30.98±9.02 and 44.17±3.96% reported by Mavi et al. (2017) for Aseel and Kadaknath chickens respectively. However, Shanmugam et al. (2014) recorded higher membrane integrity of 86-89% in 23-65 weeks old Dahlem red roosters. The differences in the membrane integrity obtained in this study and others may possibly imply that sperm membrane integrity of chickens differs by age and breeds of chickens. According to Ramu and Jeyendran (2013), membrane integrity is very important for sperm capacitation, acrosome reaction and binding of spermatozoa to the egg surface, the implication of reduced membrane integrity of spermatozoa recorded in the cocks administered 4 and 6 mgeCORT/KgBW is that, spermatozoa of highly stressed cocks will have a lower capacity to bind to the peri-vitelline membrane of egg yolk making it difficult for such sperm cell to penetrate into the germinal disc and fertilize the egg.

In the current study, administration of eCORT for 14 days significantly increased the percentage of sperm abnormality especially at higher doses (4 and 6 mgeCORT/KgBW) and this means that increased circulation of eCORT increases the sperm morphological defection. Sperm abnormalities have implication on fertility and overall reproductive performance of the animals, high proportion of spermatozoa with defective tail in an ejaculate decreases motility and fertility due to the inability of the spermatozoa to reach and penetrate an egg (Feyisa et al.*,* 2018).

Seminiferous tubular diameter and epithelial height of the testes were not affected by eCORT in this study. The values obtained for seminiferous tubules diameter for all the cocks were higher than the 206-224 μm previously reported by Orlu and Egbunike (2009) for Nigerian indigenous cocks during different seasons. However, the seminiferous tubules diameters obtained were lower than 248.56 μm reported for Barred Plymouth Rock (Orlu and Egbunike, 2009). The lack of a significant effect of eCORT on the seminiferous tubules diameters despite the decline in the semen quality means that eCORT affected the spermatozoa during storage and maturation in the efferent duct (ductus deferens and epididymis) and not during spermatogenesis in the seminiferous tubules. This further indicates that the high sperm abnormality recorded in 4 mgeCORT/KgBW and 6 mgeCORT/KgBW cocks was the secondary abnormality type occurring in the epididymis in the process of sperm ejaculation. According to Almahdi et al. (2014), sperm abnormalities that arise during spermatogenesis process in the seminiferous tubules is the primary type while secondary abnormality is that which occur in the epididymis and is not as severe as the primary type.

In birds, stressful events arising from potential predators result in increments in glucocorticoids that promote self-maintenance at an expense to reproduction (Lothery et al.*.,* 2014; Vitousek et al.*,* 2014). Results from the current study showed that eCORT had no significant influence on the mating behavior of the cocks. In Nigerian indigenous chickens, mating ratio (1C:3H, 1C:6H and 1C:9H) has no significant effect on their mating behaviors (Iyasere et al., 2020). In the current study, a mating ratio of 1C:6H was adopted to avoid the establishment of social dominance among males. A negative correlation was previously reported between sexual signal quality and CORT level (Saino et al.*,* 2002). Possible effects of CORT on expression of mating behavior are facilitated by the negative effects it has on circulating testosterone concentration (Emerson, 2001), however, in this study, circulating testosterone was also not affected differently.

Spermatogenesis in birds’ testes depends on circulating FSH, LH, and testosterone (Kirby and Froman, 2000). While some studies reported that high plasma concentration of CORT resulted in decreased testosterone concentrations and increased FSH and LH in chickens (Sahin and Kucuk, 2003, McDaniel et al.*,* 2004), there was no variation in testosterone and FSH concentration of CORT treated cocks while LH concentration was enhanced only in cocks administered with 2 mgeCORT/KgBW. Also in laying hens, heat stress caused no significant changes in FSH and LH concentration (Rozenboim et al.*,* 2007). The lack of alternation in the circulating FSH and LH of CORT treated Nigerian indigenous cocks could means that the pituitary output of gonadotropins is not affected by the dose of CORT administered and this may also be the reason why the histomorphometry of the cocks testes were also not affected since normal concentration of circulating FSH and LH enhance the development of seminiferous epithelium (Chen et al.*,* 2015) while elevated FSH concentration damages the seminiferous epithelium and spermatogenesis (Pezzella et al.*,* 2013). According to Tilbrook et al. (2000), there may be species differences in the extent to which glucocorticoids inhibit the secretion of LH and FSH and higher CORT circulation may not be the only mediators of stress suppression of FSH and LH secretion.

The level of stress imposed by eCORT was determined by measuring the H/L ratio of the cocks based on established report that H/L ratio is an indicator of stress in chickens (Gross and Siegel, 1983). H/L ratios of 0.2, 0.5 and 0.8 are characteristic of low, optimal and high degrees of stress respectively in chickens (Gross and Siegel, 1983). The H/L recorded in all the eCORT treatments in the current study was 0.5 which suggests optimal degree of stress. The plasma CORT level was not measured in the current study based on its limitations such as high variability of CORT levels, procedures such as catching and restraining the bird elevates CORT level itself if blood is not sampled within 2-3 minutes of the bird being caught (Mormède et al., 2007). Another shortcoming of blood CORT is that under chronic (prolonged) stress, CORT levels in the blood can be reduced to baseline levels by the negative feedback mechanism, which assists in the regulation of the concentration of CORT in circulation (Pariante and Lightman, 2008) such that it becomes difficult to differentiate between a chronically stressed animal and a control.

## Conclusion

This study revealed that optimal stress induced by eCORT impaired semen quality but with less impact on reproductive hormones, H/L and mating behaviors of Nigerian indigenous cocks. Also, eCORT affected spermatozoa of Nigerian indigenous cocks during maturation in the efferent duct and not during spermatogenesis in the seminiferous tubules.
